# Effect of Granulocyte-Colony Stimulating Factor on Endothelial Cells and Osteoblasts

**DOI:** 10.1155/2016/8485721

**Published:** 2016-02-24

**Authors:** Xi Ling Liu, Xiang Hu, Wei Xin Cai, Weijia William Lu, Li Wu Zheng

**Affiliations:** ^1^Discipline of Oral Diagnosis and Polyclinics, Faculty of Dentistry, The University of Hong Kong, Hong Kong; ^2^Discipline of Oral and Maxillofacial Surgery, Faculty of Dentistry, The University of Hong Kong, Hong Kong; ^3^Department of Oral and Maxillofacial Surgery, Guanghua School and Research Institute of Stomatology, Sun Yat-sen University, Guangzhou 510000, China; ^4^Department of Orthopedics and Traumatology, The University of Hong Kong, Hong Kong

## Abstract

*Objectives*. Some animal studies showed that granulocyte-colony stimulating factor (G-CSF) provides beneficial environment for bone healing. It has been well documented that endothelial cells and osteoblasts play critical roles in multiple phases of bone healing. However, the biological effects of G-CSF on these cells remain controversial. This study aimed to investigate the influence of G-CSF at various concentrations on endothelial cells and osteoblasts.* Materials and Methods*. Human umbilical vein endothelial cells (HUVECs) and human osteoblasts (hOBs) were treated with G-CSF at 1000, 100, 10, and 0 ng/mL, respectively. The capacity of cell proliferation, migration, and tube formation of HUVECs was evaluated at 72, 8, and 6 hours after treatment, respectively. The capacity of proliferation, differentiation, and mineralization of hOBs was evaluated at 24 hours, 72 hours, and 21 days after treatment, respectively.* Results*. HUVECs treated with 100 and 1000 ng/mL G-CSF showed a significantly higher value comparing with controls in migration assay (*p* < 0.001, *p* < 0.01, resp.); the group treated with 1000 ng/mL G-CSF showed a significantly lower value on tube formation. No significant difference was detected in groups of hOBs.* Conclusions*. G-CSF showed favorable effects only on the migration of HUVECs, and no direct influence was found on hOBs.

## 1. Introduction

Angiogenesis and osteogenesis are two interdependent activities which play crucial roles during bone development and regeneration [1]. Angiogenesis is the formation of new blood vessels occurring in an adult through migration and proliferation and tubular structures formation of endothelial cells. Osteogenesis takes place near newly formed vessels that mediate delivery of osteoprogenitor cells, secrete mitogens for osteoblasts, and transport nutrients and oxygen. In bone, vascular development always precedes osteogenesis [1, 2].

During skeletal development and fracture repair, the coordination of multiple events, such as migration, differentiation, and activation of multiple cell types and tissues, is required. Endothelial cells and osteoblasts are two of the major cell types involved in the process of bone regeneration. The endothelial cells make up the inner surface of the microvascular or blood vessels which supply the surrounding cells with oxygen and nutrients and remove waste products. The development of a microvasculature and microcirculation is critical for the homeostasis and regeneration of living bone, without which the tissue would simply degenerate and die [3]. Inadequate bone vascularity is associated with decreased bone formation and bone mass. Compromise of angiogenesis during fracture repair usually results in the formation of fibrous tissue. Under the circumstance of a well-developed network, the osteoblasts produce osteoid, calcify and differentiate to osteocytes, and exhibit healthy bone formation. A poor blood supply is therefore considered as a risk factor for an impaired bone healing. Factors that can stimulate the activity of endothelial cells (angiogenesis) as well as that of osteoblasts (bone formation) are likely to have a clear advantage to enhance bone repair.

Granulocyte-colony stimulating factor (G-CSF) is a clinical available growth factor, which stimulates the production of white blood cells, particularly granulocytes [4]. G-CSF also mobilizes hematopoietic stem cells into peripheral blood [4]. G-CSF has been shown to promote bone repair in animal models. It was suggested that locally applied G-SCF contributes to an ideal local environment for bone healing by supplying adequate blood flow and stimulating osteogenesis [5]. However, the effect of G-CSF on endothelial cells and osteoblast are rarely investigated, and the results remain controversial. Bussolino et al. found that G-CSF was capable of inducing the migration and proliferation of endothelial cells at 100 ng/mL [6], while Lee et al. reported that G-CSF promotes endothelial migration and tube formation at a low dose, though no effect was found on proliferation [7]. Christopher and Link suggested that long-term systematic administration of G-CSF induced apoptosis in osteoblast and inhibited its differentiation* in vivo* [8]. 

In this study, to further understand the effect of G-CSF on endothelial cells and osteoblasts, we tested G-CSF at various doses on human umbilical vein endothelial cells and human osteoblasts.

## 2. Materials and Methods

### 2.1. Reagents

Recombinant human G-CSF (rhG-CSF) purchased from GIBCO® Invitrogen (Camarillo, CA, USA) was prepared according to manufactures instruction. Human umbilical vein endothelial cells (HUVECs), endothelial cell medium (ECM), human osteoblasts (hOBs), and osteoblast medium (ObM) were purchased from ScienCell Research Laboratories (San Diego, CA, USA).

### 2.2. HUVECs

#### 2.2.1. Culture of HUVECs

HUVECs were cultured in ECM at 37°C with 5% CO_2_. Passages three to six were used in all experiments.

#### 2.2.2. Proliferation Assays

To assess the influence of rhG-CSF on HUVECs growth, MTT [3-(4,5-dimethylthiazol-2-yl)-2,5-diphenyltetrazolium bromide] cell proliferation assay was performed. All samples were run in triplicate in 48-well plates. Briefly, HUVECs were plated at 2 × 10^3^ cells/well and grown 24 hours in ECM. Medium was subsequently removed and replaced with ECM, or ECM with rhG-CSF at 10 ng/mL, 100 ng/mL, or 1000 ng/mL. The cells were incubated for 72 hours. Then medium was removed and rinsed with phosphate-buffered saline. MTT dye solution was added and cells were incubated at 37°C. After 4 hours, SDS-HCL solution was added and kept in incubator at 37°C overnight. Cells were then subjected to a spectrophotometer (SpectraMax M2, Sunnyvale, CA, USA) for measurement (*λ* = 570 nm). Level of proliferation of HUVECs was calculated as follows:

(1) $$\begin{array}{c} \text{Proliferation}\left[\;\%\;\right]=\frac{\left(\text{Absorbance in tested sample}\right)-\left(\text{Absorbance in negative control}\right)}{\left(\text{Absorbance in control}\right)-\left(\text{Absorbance in negative control}\right)}\times 100\%, \end{array}$$

where “control” denotes cells without any additives and “negative control” means an empty well without cell [9, 10].

#### 2.2.3. Wound Healing Assay

To assess the effects of rhG-CSF on the migration ability of HUVECs, wound healing assay was conducted as described by Lam et al. [9]. All samples were run in triplicate in 24-well plates. HUVECs were seeded at 1.5 × 10^4^ cells/well with ECM and incubated overnight at 37°C with 5% CO_2_. After incubation, cells were then starved for 24 hours by low serum (0.5% FBS). Then monolayers were horizontally scratched using a P100 pipette tip to create the wound. Cells were washed with phosphate-buffered saline to remove the debris. ECM alone or ECM with rhG-CSF at 10 ng/mL, 100 ng/mL, or 1000 ng/mL was added to allow for wound healing. Immediately after the change of medium, three randomly selected views along the scraped line were photographed on each well at 50x magnifications. After 8 hours of incubation, another set of images was taken using the same method.

Image analysis for signs of migration was performed using Leica Qwin Image Processing & Analysis Software V.2.6 (Leica, Cambridge, UK). The increment of migration was calculated by subtracting the length of scraped line at 8 hours from that of 0 hours. A reduction in the scraped area indicates a sign of migration.

#### 2.2.4. Tube Formation Assay

The effects of rhG-CSF on HUVEC differentiation were examined by* in vitro* tube formation on growth factor reduced Matrigel matrix (BD Biosciences, Franklin lakes, NJ). Matrigel solution was thawed overnight at 4°C, and all plasticware was precooled at −20°C. Resuspended HUVECs in ECM alone or ECM with rhG-CSF at 10 ng/mL, 100 ng/mL, or 1000 ng/mL were plated (1.5 × 10^4^/well) on growth factor reduced Matrigel (60 *μ*L/well) in a 96-well tissue culture-treated plate, as suggested by Arnaoutova et al. [11]. All samples were run in triplicate. After 6 hours of incubation at 37°C in humidified air with 5% CO_2_, endothelial network formation was examined. Images were taken at randomly chosen fields in each well at 100x magnifications. The mean value of total tubule length in each group was quantified by Leica Qwin Image Processing & Analysis Software V.2.6 (Leica, Cambridge, UK). Tube-like structures were defined as endothelial cord formations that were connected at both ends [9].

### 2.3. hOBs

#### 2.3.1. Cultures of hOBs

hOBs were cultured in ObM and incubated at 37°C with 5% CO_2_. Passages three to six were used in all experiments.

#### 2.3.2. Proliferation Assays

To assess the influence of rhG-CSF on hOBs growth, MTT cell proliferation assay was adopted. All samples were run in triplicate in 48-well plate. Briefly, 2 × 10^3^ cells were plated and grown for 24 hours in ObM. Medium was then removed and replaced with ObM, or ObM with rhG-CSF at 10 ng/mL, 100 ng/mL, or 1000 ng/mL. The cells were incubated for 72 hours. Then medium was removed and rinsed with PBS. MTT dye solution was added and cells were incubated at 37°C. After 4 hours, SDS-HCL solution was added and kept in incubator at 37°C overnight. Cells were then subjected to a spectrophotometer (SpectraMax M2, Sunnyvale, CA, USA) for measurement (*λ* = 570 nm). Level of proliferation of hOBs was calculated as described previously in Section 2.2.

#### 2.3.3. Differentiation Assay

Alkaline phosphatase (ALP) activity was evaluated as an early marker for osteogenic differentiation. Briefly, hOBs were treated with control medium and different dosages of rhG-CSF, and then ALP released in the medium was examined. All samples were run in triplicate in 24-well plate. Cells were plated at a density of 2 × 10^4^ cells/well. After overnight incubation, medium was changed with ObM alone or ObM with rhG-CSF added at concentrations of 10 ng/mL, 100 ng/mL, and 1000 ng/mL. After 72 hours of incubation, the cultures were rinsed twice with ice-cold PBS, solubilized in Tris/glycin/Triton buffer. ALP released in the medium after 72 h after treatment was assayed utilizing the p-nitrophenyl phosphate (pNPP) ALP assay kit according to the manufacturer's introduction (AnaSpec, Fremont, CA, USA). The colour change was measured spectrophotometrically at 405 nm (SpectraMax M2, CA, USA). The ALP released by the cells was normalized per microgram of cell protein. Protein content was measured in cell lysate by bicinchoninic acid (BCA) protein assay reagent kit (Pierce Biotechnology, Rockford, IL, USA) [12].

#### 2.3.4. Mineralization Assay

Mineralization of the extracellular matrix was used as a late marker of* in vitro* bone formation. All samples were run in triplicate in 24-well plates. hOBs were seeded at 4 × 10^4^ cells/well and cultured in ObM. After 24 hours, medium was switched to calcification medium [ObM also containing 10 mM *β*-glycero phosphate (Sigma) and 50 mg/mL L-ascorbic acid (Sigma)] alone, or calcification medium with rhG-CSF added at concentrations of 10 ng/mL, 100 ng/mL, or 1000 ng/mL. The medium in each group was replaced every 4 days. Cells cultured in calcification medium have been used as a basal control. After an incubation period of 21 days, the mineralized matrix was stained for calcium by Alizarin-red (ARS) staining. The cultures were fixed by covering with 4% paraformaldehyde and incubated at room temperature for 15 minutes. After rinsing with distilled water 3 times, ARS solution was added and incubated at room temperature for 20 minutes. The excess dye was removed by washing 4 times with deionized water. Then, 1 mL of water was added to each well to prevent the cells from drying, and the images were acquired. To quantify the ARS staining, 10% acetic acid was added to each well and incubated for 30 minutes. Then, the cells and acetic acid were vortexed for 30 seconds and heated to 85°C for 10 minutes. After cooling, the slurry was centrifuged at 20,000 g for 15 minutes, and the supernatant was collected. The standard/sample was added to a transparent-bottom 96-well plate, and optical density was measured at 405 nm with spectrophotometry.

### 2.4. Statistics

Normality and homogeneity of variance were checked. One-way ANOVA was performed to determine the levels of significant differences with Bonferroni post hoc tests (SPSS V.20). All statistical tests adopted significance level of *p* < 0.05.

## 3. Results

### 3.1. Effect of rhG-CSF on HUVECs

#### 3.1.1. Cell Proliferation

**Figure 1:**
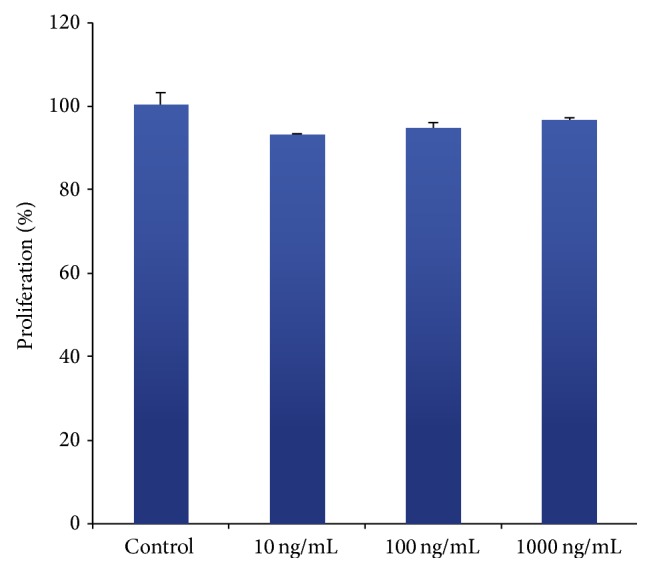
Effect of rhG-CSF on the proliferation of HUVECs. The amount of cells was determined using the MTT assay as described in Section 2. Results are expressed as percentages of the control group. The data shown are presented as mean ± SE.

All three treatment groups showed a slightly lower proliferation rate than the control group (Figure 1). No significant differences were found.

#### 3.1.2. Cell Migration

**Figure 2:**
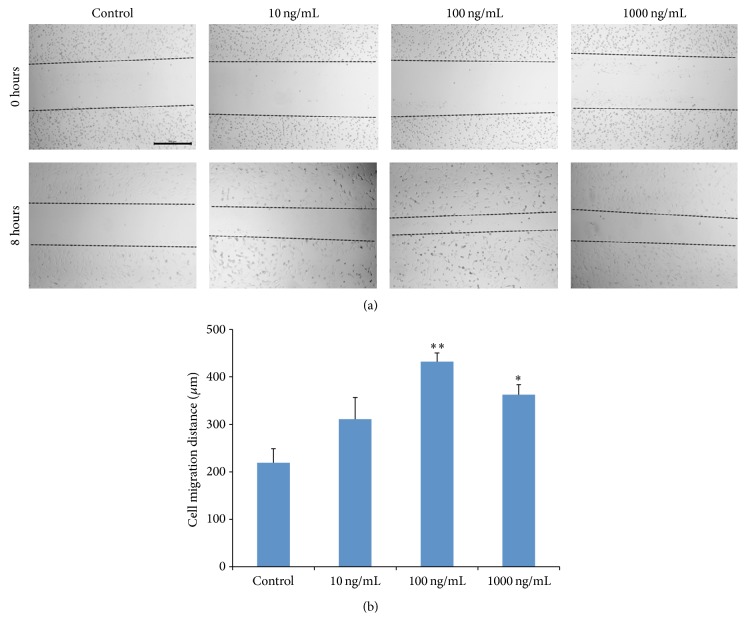
Effect of rhG-CSF on cell migration of cultured HUVECs. (a) Wound healing assay. Representative images of wound healing at the beginning (0 hours) and 8 hours after wound scratch. (b) The level of cell migration into the wound scratch was quantified by measuring the wound healing distance. Scale bar represents 500 *μ*m. The data shown are presented as mean ± SE. *∗∗* denotes *p* < 0.001, and *∗* denotes *p* < 0.01.

The effect of rhG-CSF on cell migration of HUVECs was determined by wound healing assay (Figure 2). At 8 hours after scratch, little migration was observed in the control group, whereas an obvious increment in migration was found in all treatment groups. With the raise in dosage of rhG-CSF, the increment of migration appeared to be bell-shaped. In the group treated with 100 ng/mL and 1000 ng/mL of rhG-CSF, significantly higher values of cell migration than of the control group were found (*p* < 0.001 and *p* < 0.01, resp.).

#### 3.1.3. Tube Formation

**Figure 3:**
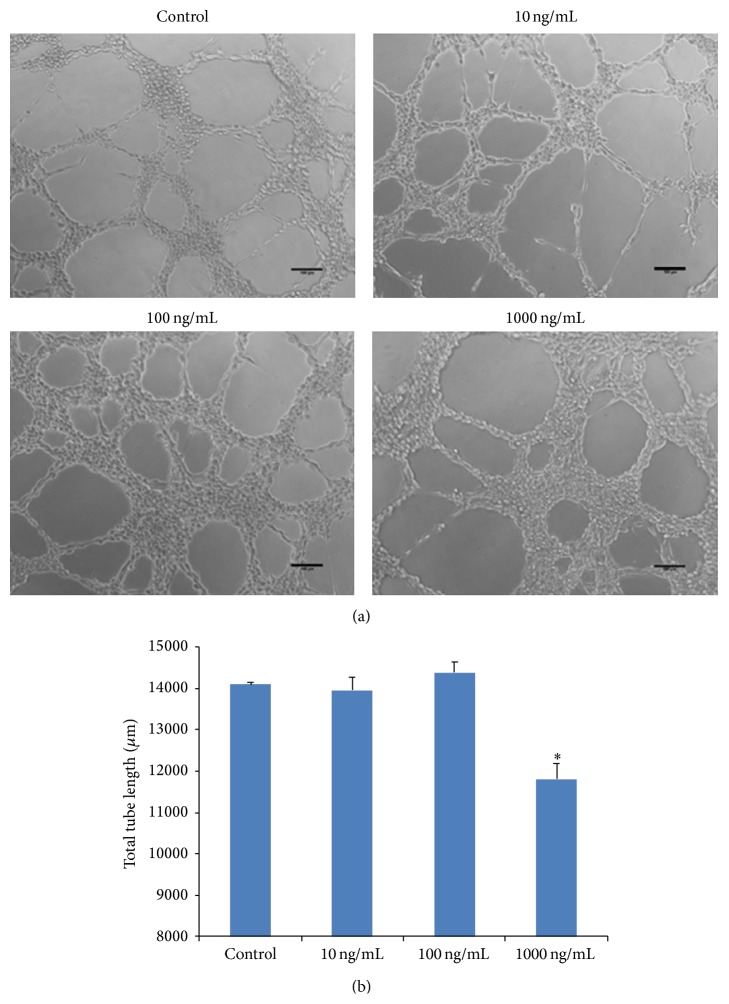
Effect of rhG-CSF on tube formation of cultured HUVECs. (a) Tube formation assay. Representative images of cultured HUVECs after incubation for 6 hours, on Matrigel-coating with rhG-CSF at indicated concentrations. (b) The length of total tube length was quantified. The data shown are presented as mean ± SE. Scale bar represents 200 *μ*m. *∗* denotes *p* < 0.01.

The processes of angiogenesis typically consist of proliferation and alignment to form tubular structures [11]. To test the ability of rhG-CSF on the induction of capillary tube formation, a tube formation model was used by culturing HUVECs on growth factor reduced Matrigel (Figure 3). Among the four groups, the groups treated with 10 and 100 ng/mL rhG-CSF showed similar values as the control group, with the 100 ng/mL group demonstrating a slightly higher value, but no statistical difference was detected, while the 1000 ng/mL rhG-CSF group showed significantly lower value in total tube length than the control group (*p* < 0.01), indicating compromised ability of tube formation.

### 3.2. Effect of rhG-CSF on hOBs

#### 3.2.1. Cell Proliferation

**Figure 4:**
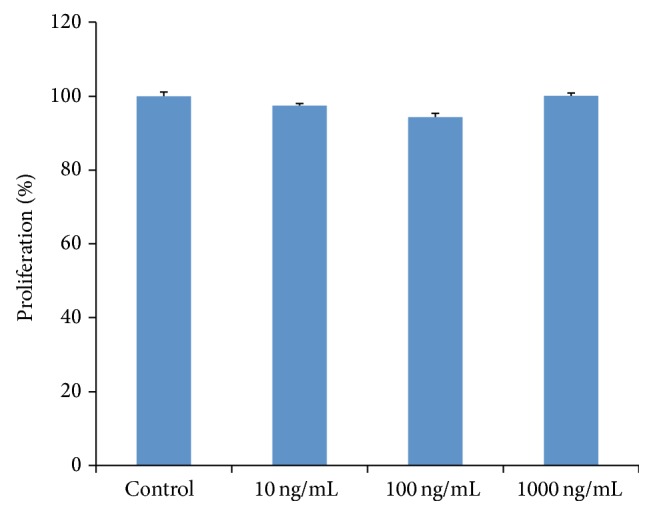
Effect of rhG-CSF on the proliferation of hOBs. The number of cells was determined using the MTT assay as described in Section 2. Results are expressed as percentages of the control group. The data shown are presented as mean ± SE.

hOBs treated with 1000 ng/mL rhG-CSF showed similar proliferation rate as the control group, while the other two groups showed slightly lower rates (Figure 4). No significant differences were found between rhG-CSF treated groups and the control group.

#### 3.2.2. Cell Differentiation

**Figure 5:**
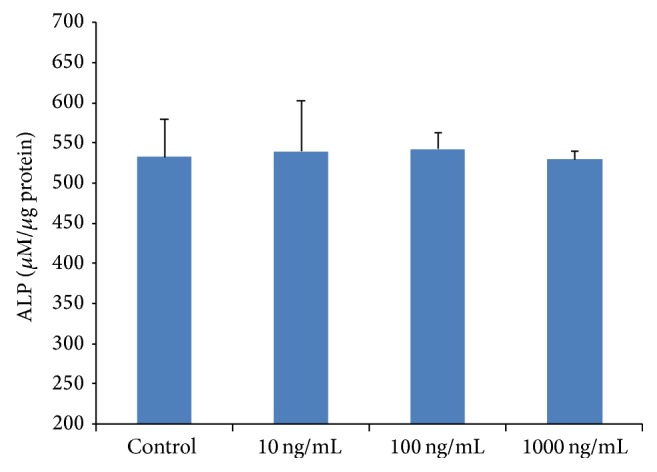
Assessment of ALP activity of hOBs at 72 hours after treatment. Results are expressed as *μ*M/*μ*g protein. The data shown are presented as mean ± SE.

Seventy-two hours after treatment, the 10 and 100 ng/mL rhG-CSF groups showed slightly higher value of ALP when compared to the control group, while the value in the group of 1000 ng/mL rhG-CSF group was lower (Figure 5). No statistical difference was detected between rhG-CSF treated groups and the control group.

#### 3.2.3. Cell Mineralization

**Figure 6:**
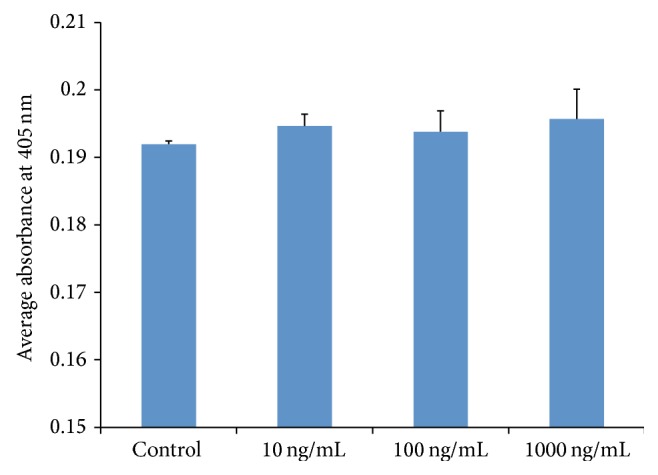
ARS staining of hOBs at 21 days of osteogenic differentiation. ARS acid extraction was used to semiquantify the production of mineral by hOBs. The data shown are presented as mean ± SE.

A quantitative ARS method was performed to detect calcium compounds deposited in the extracellular matrix as a result of mineralization. After 21-day culture in the presence of osteogenic supplements, no obvious difference of staining was observed between groups. The rhG-CSF groups showed slightly higher absorbance than the control group, with no statistical difference detected between rhG-CSF treated groups and the control group (Figure 6).

## 4. Discussion

G-CSF has been widely used to mobilize CD34+ HSCs and to increase circulating granulocytes in patients receiving bone marrow transplantation or chemotherapy [13]. It is reported that G-CSF showed the effect of recruiting HSCs and the other lineage cells—EPCs, facilitating postnatal tissue regeneration in the cardiovascular system [14]. Both in animal models and in clinical trials, G-CSF has demonstrated its angiogenic potentials as a growth factor used for stem and progenitor cell mobilization in malignant and nonmalignant disease [15, 16]. Therefore, G-CSF administration has been considered as a promising method for therapeutic angiogenesis.

Early in the 1990s,* in vitro* studies have shown that G-CSF (100 ng/mL) had direct stimulatory actions on mature vascular endothelial cells, by showing positive effect on cell migration, proliferation in culture, and capillary-like tube formation on Matrigel culture* in vitro* [6]. A recent study has demonstrated that G-CSF are capable of enhancing the expression of multiple cell cycle proteins of endothelial cells and preventing cell death by increasing cell viability, decreasing apoptosis and caspase-3 activity [17]. Besides the potential of G-CSF on angiogenesis, recent studies have further demonstrated its ability of mobilizing mesenchymal stem cells (MSC), from which osteoblasts were derived, into peripheral blood [18]. An animal study also demonstrated its potential in bone regeneration by supplying adequate blood flow and stimulating osteogenesis through local administration [5]. These findings suggest that by local administration of lower doses, G-CSF may provide a beneficial environment for angiogenesis and osteogenesis. However, controversial results are also reported. In an* in vitro* study, Tura et al. reported that G-CSF at 100 ng/mL exhibited an inhibitory effect on tube formation in HVUECs [19]. Another study reported that G-CSF induced osteoblast apoptosis and inhibited osteoblast differentiation in rat, when administrated systematically at a relatively high dose (250 *μ*g/kg per day, for 7 days) [8].

To clarify the effect of different dosages of G-CSF on endothelial cells and osteoblasts, the present study evaluated the influence of G-CSF on HUVECs and hOBs of three concentrations.

The angiogenic process of endothelial cells involves cell proliferation, migration, alignment, and tube formation [20]. In earlier studies, Bussolino et al. demonstrated that G-CSF at low dosage (10 and 100 ng/mL) stimulated angiogenic functions of mature endothelial cells, resulting in enhanced migration, proliferation, and tube formation* in vitro* [6]. However, in our study, G-CSF showed no positive effect on proliferation of HUVECs regardless of the dosage, which is consistent with the result of Lee et al. and Staško et al. [7, 21]. In wound healing assay, we found a significant enhancement on cell migration in cells treated with G-CSF at 100 ng/mL and 1000 ng/mL compared to the other group. Our findings demonstrate that G-CSF is capable of inducing migration of mature human endothelial cells. Proliferation and migration are both important early signs of angiogenesis of endothelial cells. The discrepancy found in proliferation assay compared with Bussolino et al. may be a result of the application of different derivatives of rhG-CSF [6]. As it is reported there were about one hundred derivatives of rhG-CSF created by various gene mutation techniques, and two different derivatives of rhG-CSF could result differently in cell proliferation assay [22].

Based on the differentiation of endothelial cells, tube formation assay replicates many steps in angiogenic process, including cell adhesion, migration, protease activity, alignment, and tube formation [11]. It has been reported that low concentration of G-CSF demonstrated a favorable effect on proliferation and migration through certain pathways [17, 23]. However, the mechanism of the interaction during tube formation remains unclear. In the tube formation assay, we found that 100 ng/mL of G-CSF showed higher value in the total tube length than the other groups, while 1000 ng/mL of G-CSF showed a significant lower value when compared to the control group, indicating an inhibitory effect on angiogenesis. It would be interesting to further explore the underlying mechanism of the inhibition effect at high concentration in tube formation process.

The role of osteoblast in bone tissue regeneration is characterized by three sequential stages: proliferation, differentiation, and mineralization of the extracellular matrix, which indicates the endpoint of osteoblast phenotypic expression [24]. To evaluate the three stages in osteogenesis, proliferation assay, ALP, and ARS test were performed on hOBs. The results showed that G-CSF had little effect on the proliferation and differentiation on hOBs. Though the values of G-CSF groups in the ARS staining test were slightly higher than that of the control group, no statistic differences were detected, and no dose-dependent tendency was observed. These results indicate that G-CSF alone may have limited effect on hOBs. Moreover, there have been controversies regarding the effect of G-CSF on osteoblasts under different environment. It was reported that under normal condition, a high dose of daily injection of G-CSF (250 *μ*g/kg per day, for 7 days) induced osteoblasts apoptosis and inhibited osteoblasts differentiation in a mouse model [8]. However, under fracture environment, a local administration of G-CSF at low dosage (5 *μ*g/rabbit) mobilized more osteoblasts into the defect area and resulted in enhanced osteogenesis [5]. Bone regeneration, especially during bone reconstruction, is a complex process that involves a large number of growth factors and cytokines for its regulation. In the present study, the results suggested that the effect of G-CSF on hOBs may not totally depend on the direct local effect.

## 5. Conclusion

In conclusion, these findings indicated that when applied at 100 ng/mL in HUVECs, G-CSF significantly stimulated the cell migration, which was a crucial process during angiogenesis. However, G-CSF showed little direct effect on hOBs regardless of the concentration. Further investigation is needed to evaluate a detailed mechanism, such as how high concentration of G-CSF inhibits the tube formation of HUVECs and how hOBs is stimulated by G-CSF.
